# Prevalence of oral mucosal lesions and associated risk factors in a Norwegian adult population – the HUNT4 Oral Health study

**DOI:** 10.1186/s12903-025-06162-4

**Published:** 2025-07-04

**Authors:** Thomas R. Klimowicz, Astrid J. Feuerherm, Wenche Moe Thorstensen, Lars M. Berg, Håkon Valen, Rune Becher, Abhijit Sen

**Affiliations:** 1 Center for Oral Health Services and Research Mid-Norway (TkMidt), Trondheim, Norway; 2 Department of Neuromedicine and Movement Science, Norwegian University of Science and Technology (NTNU), Trondheim, Norway; 3 Department of Public Health and Nursing, Faculty of Medicine and Health Sciences, Norwegian University of Science and Technology (NTNU), Trondheim, Norway; 4 Department of Otolaryngology, Head and Neck Surgery, St Olavs University Hospital, Trondheim, Norway; 5 Department of Clinical Dentistry, Faculty of Health Sciences, The Arctic University of Norway (UiT), Tromsø, Norway; 6 Department for Air Quality and Noise, Norwegian Institute of Public Health (NIPH), Oslo, Norway; 7 Nordic Institute of Dental Materials (NIOM), Oslo, Norway; 8 Department of Maxillofacial Surgery, St. Olavs University Hospital, Trondheim, Norway

**Keywords:** Oral mucosal lesions, Sociodemographic, Risk factors, Prevalence

## Abstract

**Background:**

Most studies on the prevalence of oral mucosal lesions (OMLs), including those from Norway, focus on patient populations. This study aimed to estimate the overall prevalence of OMLs and its types across different age groups and to examine their association with selected risk factors in a general adult population of Norway.

**Methods:**

This cross-sectional study included a random sample of 4,909 individuals aged 19 years and older who completed questionnaires and underwent a standardized clinical examination for OMLs in the HUNT Oral Health Survey (2017–2019). The classification of OMLs was based on the World Health Organization and NHANES III guidelines. Odds Ratio (OR) with 95% confidence intervals (CI) were calculated using multivariable logistic regression to assess associations between OMLs and potential risk factors, adjusting for relevant confounders.

**Results:**

The overall prevalence of OMLs was 7.5%, with a higher proportion observed in individuals aged 60 and older. The most prevalent oral lesions were exophytic lesions (3.1%) and white lesions (1.5%). Notably, individuals with a low/middle level of education had higher odds of having an OMLs than those with a higher level of education (OR, 1.27, 95% CI: 1.00-1.61) in the fully adjusted model. Exploratory analyses of lesion types found a positive association between smoking status and white lesions (OR, 1.77, 95% CI:1.05–2.97), as well as between a history of cancer and red-blue lesions (OR, 2.17, 95% CI, 1.14–4.11).

**Conclusion:**

In this population-based study, 7.5% of Norwegian adults had one or more OMLs, with higher prevalence in participants aged 60 years and older. Further research is needed to confirm these findings in similar populations.

**Supplementary Information:**

The online version contains supplementary material available at 10.1186/s12903-025-06162-4.

## Introduction

Oral mucosa may serve as a mirror to an individual’s overall health [1]. Any alterations in appearance, color, texture, or integrity of the oral mucosal surfaces could be classified as an oral mucosal lesion [2, 3], making them a heterogeneous group of disorders. Some oral lesions can negatively impact oral health-related quality of life by making eating difficult, hindering oral hygiene, interfering with swallowing and speaking, and impacting overall well-being [1, 4, 5]. Therefore, early detection and timely treatment of certain OMLs are essential to prevent complications and maintain proper oral function [6].

Several studies have reported the prevalence of OMLs, with global rates ranging from 5 to 65% [7]. These variations across studies are largely due to geographical, genetic, cultural and sociodemographic differences, along with factors like lifestyle choices, healthcare access, and oral behaviors [3, 4, 8]. Furthermore, a systematic review from 2019 highlights methodological weaknesses in OMLs prevalence studies, including inconsistent definitions and subgrouping, small sample sizes, low response rates, lack of inter-rater reliability, and absence of confidence intervals, making cross-study comparisons challenging [7].

Older age [4, 9], high alcohol intake, and smoking have been associated with a higher prevalence of OMLs [3, 10]. Notably, potentially precancerous lesions such as oral lichen planus, leukoplakia, and erythroplakia are particularly linked to alcohol and tobacco use [3]. Other contributing factors might include oral- or systemic infections, systemic illnesses, underlying chronic diseases, and local trauma [4, 11]. Also, low socioeconomic status and health disparities have been reported to be associated with a higher prevalence of OMLs [12, 13].

Many studies on OML prevalence has focused on the prevalence within specific patient populations [14–16] or specific age groups [17–19]. There are limited studies from Scandinavian countries [20, 21] and only one study from Norway that has reported OML prevalence [20], and it was based on patients visiting dental clinics.

Therefore, this cross-sectional study aimed at estimating the overall prevalence and distribution of OMLs and it’s types across different adult age groups in Norway. Additionally, this study assesses the association between OMLs and selected risk factors of clinical relevance in a general adult Norwegian population.

## Materials and methods

### Study design and study sample

**Fig. 1:**
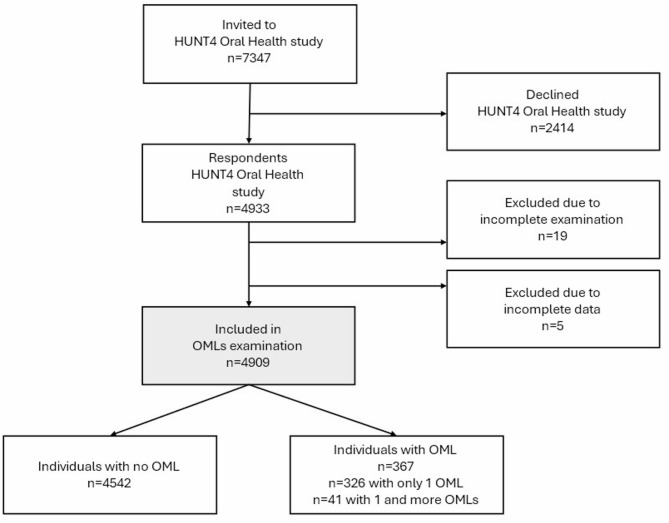
Flow chart of the study population

The Trøndelag Health study (the HUNT study) is a large population-based longitudinal study of the adult population [22, 23]. During the fourth phase of the HUNT survey (2017-19), a total of 7347 individuals aged 19 and older were randomly selected and invited to participate in the HUNT4 Oral Health study. Of these, 4933 individuals (response rate 67.1%) were enrolled and underwent a comprehensive, standardized oral health assessment, including radiographic and oral mucosal lesion examinations [24, 25]. Due to incomplete examination or registration, 24 individuals were excluded from the study. In total, 4909 individuals were included in this study (Fig. 1).

### Data collection and measurement

Data on sociodemographic factors (age, sex, education, household income), oral health behaviors (frequency of toothbrushing, dental care utilization), smoking, snus use (moist snuff), self-reported dental- and general- health status, and history of medical conditions/diseases were collected through a structured questionnaire. In addition, we included data on C-reactive protein (CRP) level measured in mg/L, and Body Mass Index (BMI) calculated as weight (in kilograms) divided by the squared value of height (in meters).

### Oral mucosal examination

The oral health examination in HUNT4 was conducted at six field stations located in Northern Trøndelag [24, 26]. All dentists (*n* = 12), dental hygienists (*n* = 7), and dental nurses (*n* = 19) at the field stations underwent comprehensive theoretical and practical training by the specialists (TRK and LMB) at the Center for Oral Health Services and Research (TkMidt) in Trondheim, before commencing the data collection for the HUNT4 Oral Health Survey.

Oral mucosal conditions were examined for all study individuals using standardized protocols adapted from the World Health Organization (WHO) and NHANES III (Third National Health and Nutrition Examination Survey) [2, 27]. During the data collection, OMLs were defined as any visible and/or palpable findings that deviated from normal or healthy mucosa. All lesions were recorded on a study form, with an annotation of the lesion’s characteristics (location, size, borders, surface, and reported duration). All observed lesions with the corresponding forms were also photographed for documentation and later reassessment as part of the quality control process.

For the purpose of this study, we chose to exclude all oral mucosal lesions attributed to well-established underlying local causes, such as dental infections, amalgam tattoos (normal iatrogenic findings), superficial inflammation related to dental prostheses, or normal anatomical variations (like exostoses such as torus mandibularis or torus palatinus). Additionally, lesions resulting from recent surgical treatments, chemotherapy, radiotherapy, or local superficial trauma at the application site of snus were excluded from evaluation to ensure that this study focuses on lesions with partially known or unknown etiology.

### Clinical image evaluation

A specialist in oral surgery (TRK) evaluated the intraoral findings and photos based on WHO guidelines [2] and the Pocket Atlas of Oral Diseases [28]. Tentative diagnoses of OMLs were classified according to the ICD-10 codes catalog; however, no verification through histopathological examinations or supplementary hematological tests was conducted. When necessary, radiological images were utilized to support the evaluation of OMLs. For validation and quality control, a specialist in multidisciplinary dentistry (LMB) was consulted as needed for reassessment in cases of uncertainty during TRK’s clinical assessment of images. Overall, 39.7% of intraoral findings were reassessed by LMB to ensure data reliability. An agreement was reached in all cases.

### Outcome

The defined outcome of interest was the presence of OMLs (yes, no) in the oral cavity. Each type of OMLs was recorded only once per individual, regardless of its occurrence at multiple locations. Using a combination of photo-based data and clinical information, the OMLs were categorized into seven clinical subgroups adapted from textbooks [28, 29] defined as exophytic lesions: D10.30, K06.8, K11.6, Q38.6, D17.0; white lesions: K13.2, L43.9, K13.1; red-blue lesions: D18.09, D18.01; red lesions: K13.2, L12.1, K13.0; tongue lesions: K14.3, K14.5, K14.1, K14.2; ulcerative lesions: B00.1, K12.0, K12.30 and other alteration: L81.4, D22.0, L57.9.

### Exposure(s)

Several risk factors such as education level, household income, visits to the dentist during the last 24 months, smoking, snus use, BMI levels, dental health status, general health status, history of diseases such as cancer, cardiovascular diseases (CVD), rheumatic diseases, respiratory diseases, psychiatric disorders, endocrine diseases, and multimorbidity (number of chronic diseases) which were of clinical relevance were considered for the analyses.

Education level, originally grouped in six categories from the questionnaire, was condensed into three groups: ≤ 10 years including compulsory school (low), 11–13 years including secondary school/vocational training, both started or completed (middle), and ≥ 14 years including college/university, both started or completed (high, reference group) [24, 25]. For regression analyses, low and middle level education group were pooled.

Self-reported annual household income before taxation, recorded in Norwegian Kroner (NOK), was grouped into five categories (< 250,000 (very low), 250,000–450,000 (low), 450,001–750,000 (middle), 750,001–1,000,000 (high), and > 1,000,001 (very high, reference group).

Visits to the dentists during the last 24 months of dental check-ups were categorised as ‘no’ and ‘yes’.

The original smoking and snus use variable (never, former daily, former occasionally, current daily, current occasionally) collected at HUNT4 were each dichotomized into two groups: never (reference group) and ever (incl. former and current daily or occasionally users for both smokers and snus users).

BMI was categorised according to WHO guidelines as < 18.5 kg/m² (underweight), 18.5–24.9 kg/m² (healthy normal), 25.0–29.9 kg/m² (overweight), and ≥ 30 kg/m² (obese). For the regression analysis, the underweight individuals were excluded due to the small number of cases in this category.

The self-reported general health was categorized as very good (reference group), good, not so good and poor, and self-reported dental health status (“How would you say your dental health is?”) was categorized as very good (reference group), good, bad, and very bad.

Individuals with a self-reported history of cancer, CVD (defined as having history of angina, heart attack, heart failure, atrial fibrillation, and/or stroke), rheumatic diseases (defined as having history of psoriasis, arthritis, and/or ankylosing spondylitis), respiratory diseases (including history of asthma and/or chronic obstructive pulmonary disease (COPD)), psychiatric diseases, endocrine diseases (with history of diabetes and/or thyroid dysfunction) were coded as 1, and those with no such disease history were coded as 0 (reference group). Each of these diseases was assessed independently.

Multimorbidity (i.e., the number of chronic diseases) was categorized into three groups: none (reference), 1, and ≥ 2 chronic diseases mentioned above.

### Covariates

Age (in years, continuous), sex (female, male), frequency of toothbrushing (seldom or never, once per week, once per day, two or more times per day; dichotomized into 0 = good (once per day and more) and 1 = bad (seldom or never, less than once per day), serum CRP (continuous, mg/L) were considered potential confounders in the analyses of associations between specific exposure of interest and the presence of OMLs.

### Statistical analysis

Descriptive statistics were used to analyse the sociodemographic, anthropometric, and clinical characteristics of individuals with and without OMLs. Categorical variables were presented as frequencies and percentages. OMLs was defined as a binary outcome (0 = absence, 1 = presence). The prevalence and 95% confidence interval (CI) of clinically diagnosed OMLs and its types were presented across different age groups (19-39y, 40-59y, 60-79y, ≥ 80y).

Multivariable logistic regression analyses were performed to assess independent association between selected exposure(s) (education, household income, visit to dentist in last 24 months, smoking, snus use, BMI level, dental health status, general health status, history of disease such as cancer, CVD, rheumatic diseases, respiratory diseases, psychiatric disorders, endocrine diseases, multimorbidity) and the presence of any OMLs as well as the four most common OML types. Due to low or insufficient statistical power, we did not explore regression analyses for OML types such as tongue lesions, ulcerative lesions, and other alterations, limiting statistical inference. While looking at the association between specific exposure of interest and outcome, two models were constructed. Model 1 was adjusted for age (as a continuous variable), while model 2 was adjusted for potential confounders identified based on knowledge from prior literature and visualized using a directed acyclic graph (DAG). Any covariates identified as mediators in the DAG between a specific exposure and the outcome were not treated as confounders and were thus excluded. Further, to assess the potential for residual confounding due to smoking, we conducted a sensitivity analysis using smoking pack-years used previously in a HUNT4 study [30]. Ever-smokers were categorized into ≤ 10 pack-years, > 10 pack-years, and no pack-years. The pack-years were calculated for daily ever-smokers based on smoking duration and intensity (number of cigarettes smoked per day). No pack-years refers to cases with either no data on smoking duration or intensity or both. These associations between exposure(s) and outcomes were computed as odds ratio (OR) with corresponding 95% CI. Statistical analyses were conducted using SPSS software (version 29.0, IBM Corporation, Armonk, NY, USA).

### Ethical considerations

Informed consent was obtained from all individuals by the HUNT Research Center and/or their legal guardians, adhering to applicable guidelines and regulations. The study adhered to the principles outlined in the Declaration of Helsinki and received approval from both the Norwegian Regional Committees for Medical and Health Research Ethics (49225/REK midt) and the Norwegian Centre for Research Data (NSD/809218).

## Results

Among the 4909 individuals included, 367 exhibited OMLs (326 had only one type of OML and 41 had one or more types of lesions). Given the possibility of multiple mucosal changes in each participant, a total of 412 oral mucosal lesions were identified. The mean age of the study population was 51.8 years (SD = 16.5) with the age range 19–94 years, and females constituted 56% of the study population (Table 1).

**Table 1:** Characteristics of the study population without and with OMLs at HUNT4 Oral Health study (2017–2019)

Characteristics	OML absence (*n* = 4542) *n* (%)	OML presence^¥^ (*n* = 367) *n* (%)
**Sex**		
Female	2551 (56.2)	194 (52.9)
Male	1991 (43.8)	173 (47.1)
**Education level** ^a^		
Low	328 (7.2)	32 (8.7)
Middle	2117 (46.6)	195 (53.2)
High	2074 (45.7)	137 (37.3)
Unknown	23 (0.5)	3 (0.8)
**Household income** ^b^		
Very Low	323 (7.1)	23 (6.3)
Low	829 (18.3)	81 (22.1)
Middle	1273 (28.0)	123 (33.5)
High	1045 (23.0)	73 (20.0)
Very high	972 (21.4)	61 (16.6)
Unknown	100 (2.2)	6 (1.6)
**Visit to dentist** ^c^		
No	476 (10.5)	31 (8.4)
Yes	3241 (71.3)	292 (79.6)
Unknown	825 (18.2)	44 (12.0)
**Smoking** ^d^		
Never	2074 (45.7)	151 (41.1)
Ever	2440 (53.7)	215 (58.6)
Unknown	28 (0.6)	1 (0.3)
**Smoking (pack years)** ^d^		
Never	2074 (45.7)	151 (41.1)
Ever daily ≤ 10 pack years	710 (15.6)	60 (16.3)
Ever daily, > 10 pack years	834 (18.4)	88 (24.0)
Ever smokers, no pack-years	896 (19.7)	67 (18.3)
Unknown	28 (0.6)	1 (0.3)
**Snus use** ^e^		
Never	3377 (74.4)	280 (76.3)
Ever	1105 (24.3)	82 (22.3)
Unknown	60 (1.3)	5 (1.4)
**BMI** ^f^		
Underweight	36 (0.8)	2 (0.5)
Normal weight	1593 (35.0)	112 (30.5)
Overweight	1879 (41.4)	161 (43.9)
Obesity	1022 (22.5)	88 (24.0)
Unknown	12 (0.3)	4 (1.1)
**Dental Health status**		
Very Good	660 (14.5)	50 (13.6)
Good	2402 (52.9)	212 (57.8)
Bad	378 (8.3)	28 (7.6)
Very Bad	39 (0.9)	2 (0.6)
Unknown	1063 (23.4)	75 (20.4)
**General Health status**		
Very Good	828 (18.2)	55 (15.0)
Good	2678 (59.0)	224 (61.0)
No so Good	931 (20.5)	76 (20.7)
Poor	54 (1.2)	6 (1.6)
Unknown	51 (1.1)	6 (1.6)
**Cancer** ^g^		
No	4095 (90.2)	314 (85.5)
Yes	294 (6.5)	38 (10.4)
Unknown	153 (3.3)	15 (4.1)
**CVD** ^h^		
No	3889 (85.6)	295 (80.4)
Yes	462 (10.2)	52 (14.2)
Unknown	191 (4.2)	20 (5.4)
**Rheumatic Diseases** ^i^		
No	3880 (85.4)	302 (82.3)
Yes	466 (10.3)	51 (13.9)
Unknown	196 (4.3)	14 (3.8)
**Respiratory Diseases** ^j^		
No	3776 (83.1)	295 (80.4)
Yes	599 (13.2)	55 (15.0)
Unknown	167 (3.7)	17 (4.6)
**Psychiatric Disorders** ^k^		
No	3536 (77.9)	285 (77.7)
Yes	840 (18.5)	69 (18.8)
Unknown	166 (3.6)	13 (3.5)
**Endocrine Diseases** ^l^		
No	3857 (85.0)	307 (83.7)
Yes	506 (11.1)	43 (11.7)
Unknown	179 (3.9)	17 (4.6)
**Multimorbidity** ^m^		
0	2087 (45.9)	154 (42.0)
1	1438 (31.7)	111 (30.0)
2 ≥	621 (13.7)	69 (19.0)
Unknown	396 (8.7)	33 (9.0)

OML absence = Individuals with no lesions; OML presence = Individuals with ≥ 1 any kind of lesions

^¥^ 1 or more lesions per individual, Unknown = missing data

^a^ Education years: Distributed in three categories: years ≤ 10 (Low), years 11–13 (Middle), years ≥ 14 (High)

^b^ Household annual income total in NOK: Distributed in five categories, < 250 000 (Very Low), 250 001–450 000 (Low), 450 001–750 000 (Middle), 750 001–1 000 000 (High), > 1 000 001 (Very high)

^c^ Visit to dentist over the past 24 months (multiple visits per participant were possible)

^d^ Smoking status groups categorized as never and ever smokers. Smoking (pack years) was categorized in detail into never, ever ≤ 10 pack-years, ever > 10 pack-years, ever no pack-years, calculated for daily ever smokers based on smoking duration and consumption

^e^ Snus use categorized as never and ever snus user

^f^ BMI categories: underweight (< 18.5 kg/m²), healthy (18.5–25 kg/m²), overweight (25.0–30 kg/m²), and obesity (> 30 kg/m²)

^g^ Cancer history includes any kind of cancer

^h^ CVD (cardiovascular diseases) history includes angina or heart attack or heart failure or atrial fibrillation or stroke

^i^ Rheumatic diseases history includes psoriasis or arthritis or ankylosing spondylitis

^j^ Respiratory diseases history includes asthma or COPD

^k^ including any kind of psychiatric disorders history

^l^ Endocrine diseases history includes any kind of diabetes or any kind of thyroid dysfunction

^m^ Multimorbidity: computed form history of cancer, psychiatric disorders and disorder groups (CVD, rheumatic disease, respiratory disorders, endocrinology disease) into three categories: no (45.7% of individuals), one (31.6% of the individuals), ≥ 2 chronic diseases (14.1% of individuals) and 8.7% unknown in the study population

Individuals with one or more oral mucosal lesions were somewhat more likely to be males, have a lower level of educational attainment, belong to the lower household income group, visited the dentist more frequently, and have higher rates of overweight and obesity compared to those without such lesions. They were also more often ever smokers, never snus users and had a greater prevalence of cancer, CVD, rheumatic, and respiratory diseases. The distribution of all disease groups within the study population is provided in Supplementary Table S1.

The overall prevalence of OMLs was found to be 7.5% (95% CI: 6.7–8.2), with a higher prevalence among individuals aged 60 and older compared to younger groups (Table 2).

**Table 2:** Prevalence of OMLs across different age groups in a study population

	Age Distribution (in years)				
**Individuals with ≥ 1 OML**	*n* = 4909	**19-39y** *n* = 1280	**40-59y** *n* = 1944	**60-79y** *n* = 1522	**80-94y** *n* = 163
**Yes** ^¥^	**367** 7.5% (6.7–8.2)	**49** 3.8% (2.8–4.9)	**132** 6.8% (5.7–7.9)	**161** 10.6% (9.0-12.1)	**25** 15.3% (9.8–20.9)
**No**	**4542** 92.5% (91.8–93.3)	**1231** 96.2% (95.1–97.2)	**1812** 93.2% (92.1–94.3)	**1361** 89.4% (87.9–91.0)	**138** 84.7% (79.1–90.2)

^¥^Individuals diagnosed with OML may have one or more than one lesion

Table 3 presents the distribution of OMLs types across different age groups, showing how the prevalence of the different OMLs types differs across the age categories. Overall, exophytic lesions (3.1%) were the most common, followed by white lesions (1.5%), and red-blue lesions (1.3%). The point prevalence of exophytic, white and red-blue lesions was highest in the age groups 60 and older. Additionally, a detailed frequency distribution of all OMLs by diagnosis is presented in Supplementary Table S2.

**Table 3:** Prevalence of OMLs types across different age groups

	Age Distribution (in years)				
	*n* = 4909	**19-39y** *n* = 1280	**40-59y** *n* = 1944	**60-79y** *n* = 1522	**80-94y** *n* = 163
**Exophytic lesionsª**	**151** ^**¥**^ 3.1% (2.6–3.6)	**14** 1.1% (0.5–1.7)	**55** 2.8% (2.1–3.6)	**70** 4.6% (3.6–5.7)	**12** 7.4% (3.4–11.4)
**White lesions** ^b^	**73** ^**¥**^ 1.5% (1.2–1.8)	**10** 0.8% (0.3–1.4)	**23** 1.2% (0.7–1.7)	**33** 2.2% (1.4–2.9)	**7** 4.3% (1.2–7.4)
**Red-blue lesions** ^c^	**63** ^**¥**^ 1.3% (1-1.6)	**0**	**16** 0.8% (0.4–1.2)	**41** 2.7% (1.9–3.5)	**6** 3.7% (0.8–6.6)
**Red lesions** ^d^	**42** ^**¥**^ 0.9% (0.6–1.1)	**8** 0.6% (0.2–1.2)	**19** 1% (0.5–1.4)	**12** 0.8% (0.3–1.2)	**3** 1.8% (0-3.9)
**Tongue lesions** ^e^	**33** ^**¥**^ 0.7% (0.4–0.9)	**7** 0.5% (0.2–1.1)	**13** 0.6% (0.3-1)	**12** 0.8% (0.3–1.2)	**1** 0.6% (0-1.8)
**Ulcerative lesions** ^f^	**30** ^**¥**^ 0.6% (0.4–0.8)	**8** 0.6% (0.2–1.2)	**14** 0.7% (0.3–1.1)	**7** 0.5% (0.1–0.8)	**1** 0.6% (0-1.8)
**Other alterations** ^g^	**11** ^**¥**^ 0.2% (0.1–0.4)	**4** 0.3% (0.1–0.8)	**3** 0.2% (0-0.3)	**4** 0.3% (0.01–0.5)	**0**

^¥^ An individual can be present in one or more OML type groups

^a^ Exophytic lesions includes fibroma (D10.30) or papilloma (D10.30) or fibrous epulis (K06.8) or mucocele-oral (K11.6) or fordyce granules (Q38.6) or lipoma oral (D17.0)

^b^ White lesions includes leukoplakia (K13.2) or oral lichen planus (L43.9) or leukokeratosis nicotina palati (K13.2) or nicotine stomatitis (K13.2) or morsicatio buccarum (K13.1)

^c^ Red-blue lesions includes oral hemangiomas (D18.09) or hemangioma lip/skin (D18.01)

^d^ Red lesions includes: erythroplakia (K13.2) or mucous membrane pemphigoid (L12.1) or rhagades and cheilitis (K13.0)

^e^ Tongue Lesions includes lingua villosa nigra (K14.3) or lingua plicata (K14.5) or lingua geografica (K14.1) or median rhomboid glossitis (K14.2)

^f^ Ulcerative lesions includes herpes labialis (B00.1) or aphthous ulcerations (K12.0) or aphthous stomatitis (K12.0) or long period ulceration (K12.30)

^g^ Other Alterations includes lentigo (L81.4) or nevus- melanocytic naevi of lip (D22.0) or solar elastosis (L57.9)

In the multivariable regression analyses in Table 4, the odds of any OMLs presence were higher among individuals with low/middle level of education when compared to those with higher level of education (OR,1.27, 95% CI: 1.00-1.61) in the fully adjusted model. When examining specific OML types, we observed a positive association between ever smokers and the presence of white lesions (OR, 1.77, 95% CI:1.05–2.97), compared to never smokers. We also found an association between a history of cancer and red-blue lesions (OR, 2.17, 95%CI: 1.14–4.11). Further, no association with other exposures was observed (Table 4, Supplementary Table S3-S6).

**Table 4:** Multivariable logistic regression analyses to assess the association between selected exposure(s) and OML presence

	Age-adjusted Model 1 OR (95% CI)	Fully-adjusted Model 2 OR (95% CI)
**Education level** ^**a**^	N = 4793	N = 4793
High	1.00 (ref.)	1.00 (ref.)
Middle/Low	1.28 (1.02–1.60)	1.27 (1.00-1.61)
**Household income** ^**b**^	N = 4793	N = 4793
Very high	1.00 (ref.)	1.00 (ref)
High	1.10 (0.77–1.56)	1.04 (0.73–1.49)
Middle	1.28 (0.93–1.78)	1.19 (0.85–1.66)
Low	1.18 (0.82–1.68)	1.07 (0.73–1.55)
Very low	1.08 (0.65–1.79)	0.99 (0.59–1.68)
**Visit to Dentist** ^**d**^	N = 3956	N = 3956
No	1.00 (ref.)	1.00 (ref.)
Yes	1.02 (0.69–1.50)	1.02 (0.68–1.52)
**Smoking** ^**d**^	N = 4777	N = 4777
Never	1.00 (ref.)	1.00 (ref.)
Ever	1.08 (0.86–1.34)	1.05 (0.84–1.31)
**Snus use** ^**e**^	N = 4734	N = 4734
Never	1.00 (ref.)	1.00 (ref.)
Ever	1.26 (0.96–1.66)	1.24 (0.92–1.67)
**BMI categories**^**d**^, **Kg/m**^**2**^	N = 4744	N = 4744
Healthy	1.00 (ref.)	1.00 (ref.)
Overweight	1.09 (0.84–1.40)	1.06 (0.82–1.37)
Obese	1.12 (0.83–1.50)	1.08 (0.80–1.45)
**Dental Health Status** ^**f**^	N = 3513	N = 3513
Very Good	1.00 (ref.)	1.00 (ref.)
Good	1.02 (0.74–1.42)	0.99 (0.71–1.38)
Bad	0.75 (0.45–1.25)	0.74 (0.44–1.24)
Very bad	0.60 (0.14–2.58)	0.56 (0.13–2.43)
**General Health Status** ^**g**^	N = 4733	N = 4733
Very Good	1.00 (ref.)	1.00 (ref.)
Good	1.07 (0.78–1.46)	1.02 (0.75–1.41)
No so Good	0.91 (0.62–1.32)	0.85 (0.58–1.25)
Poor	1.61 (0.66–3.94)	1.47 (0.60–3.63)
**Cancer** ^**g**^	N = 4631	N = 4631
No	1.00 (ref.)	1.00 (ref.)
Yes	1.22 (0.84–1.77)	1.22 (0.85–1.77)
**CVD** ^**g**^	N = 4587	N = 4587
No	1.00 (ref.)	1.00 (ref.)
Yes	0.97 (0.69–1.40)	0.96 (0.68–1.35)
**Rheumatic Diseases** ^**c**^	N = 4596	N = 4596
No	1.00 (ref.)	1.00 (ref.)
Yes	1.13 (0.82–1.56)	1.17 (0.85–1.61)
**Respiratory Diseases** ^**g**^	N = 4613	N = 4613
No	1.00 (ref.)	1.00 (ref.)
Yes	1.21 (0.89–1.64)	1.19 (0.87–1.62)
**Psychiatric Disorders** ^**g**^	N = 4621	N = 4621
No	1.00 (ref.)	1.00 (ref.)
Yes	1.13 (0.86–1.49)	1.14 (0.86–1.51)
**Endocrine Diseases** ^**g**^	N = 4604	N = 4604
No	1.00 (ref.)	1.00 (ref.)
Yes	0.85 (0.61–1.20)	0.86 (0.61–1.22)
**Multimorbidity** ^**g**^	N = 4387	N = 4387
0	1.00 (ref.)	1.00 (ref.)
1	0.95 (0.73–1.23)	0.95 (0.73–1.23)
≥ 2	1.13 (0.82–1.54)	1.13 (0.82–1.55)

OR = odds ratio, CI = confidence interval

Model 2 Fully-adjusted model includes confounders (presented below) for selected exposure(s) and OML

^a^**Education level**: adjusted for age, income

^b^**Household income**: adjusted for age, education

^c^**Rheumatic diseases**: adjusted for age, sex, CRP

^d^**Visit to dentist**,** smoking**,** BMI level**: adjusted for age, sex, education, income

^e^**Snus use**: adjusted for age, sex, education, income, smoking (never, ever)

^f^**Dental health status**: adjusted for age, sex, education, income, smoking (never, ever), frequency of toothbrushing

^g^**General health status**,** Cancer history**,** CVD**,** Respiratory Diseases**,** Psychiatric Disorders**,** Endocrine Diseases**,** Multimorbidity**: adjusted for age, sex, education, income, smoking (never, ever)

In the sensitivity analysis, the results remained largely unchanged from the main analysis when adjusting for smoking pack-years (data not shown).

## Discussion

In this population-based cross-sectional study of 4909 adults from the HUNT4 Oral Health survey, we found an overall prevalence for individuals with one or more OMLs of 7.5%, when disregarding those associated with known reported cause such as fistula due to dental infection or mucosal changes due to local trauma, or iatrogenic scarring. The most common oral lesions were exophytic lesions, followed by white and red-blue lesions. The prevalence of any OMLs increased with higher age. For any OMLs, we found a low/middle level of education to be significantly associated with higher odds of having any OMLs compared to those with higher education level. However, this association was not significant for the most common OML types (exophytic lesions, white lesions, red-blue lesions, and red lesions). Exploratory analyses examining the association between different exposures and the presence of specific OML types revealed an association between ever smoker and the presence of white lesions, as well as an association between a cancer history and red-blue lesions.

The OMLs prevalence in our study closely aligns with a previous cross-sectional study from Northern Norway. The study reported an OMLs prevalence of 7.6% among 3122 adult dental patients [20] which dropped to 5.4% when the data were adjusted to match the OMLs criteria we used.

Similarly, other cross-sectional studies conducted in Sweden [21], Finland [18], Germany [9], and USA [31] reported similar prevalences of 8.5%, 10.5%, 11.8% and 9.6%, respectively, when the same criteria for OMLs as in the present study were used. The predominance of exophytic lesions observed in our study was not consistent with the previous findings from Northern Norway [20]. However, the German study, examining individuals aged 20–81 years, reported a predominance of exophytic and white lesions similar to the present study [9]. Contrary to the present study, another study from Germany [32], comparing two distinct age groups, 35–44 and 65–74 years reported tongue-related OMLs as more common in the older age group [32]. This may suggest potential differences in study populations, methodologies, or regional factors influencing OMLs distribution. In line with our study, the Swedish study also reported very few cases of mucous membrane pemphigoid, pemphigus lesions, or malignant lesions. However, the Swedish study did not explore age-specific variations, limiting insights into which age groups where OMLs were most prevalent [21]. Our finding of an association between smoking and white lesions (for example, leukoplakia), is consistent with findings from others studies [15, 33]. The observed association between history of cancer and red-blue lesions is speculative, as there is no clear clinical explanation. Both cancer and OMLs may result from complex interplay of confounders like BMI, income, education, smoking, and age, considered in the regression model. Therefore, the positive association between cancer history and red-blue lesion may reflect the combined influence of these factors. Additionally, residual confounding could play a role, as unmeasured or inadequately controlled factors may influence this association. Given the small sample size of individuals with history of cancer and red-blue lesions, this finding could also be a chance occurrence. Similarly, the inverse association observed between education level and any OMLs may indicate that education acts as a proxy for multiple underlying risk factors rather than an independent determinant.

The present study has several strengths. First, data on OMLs were collected through a standardized clinical examination conducted by trained public dental healthcare personnel. Examiners were trained to distinguish, classify, and record lesions in a consistent manner. Further, the findings were reviewed using clinical images, relevant clinical information of the individuals, and radiological data (if needed) by two dentists with relevant clinical specialities before making the final diagnostic decision. The combination of image-based and clinical examination data enhances diagnostic accuracy, a practice employed by a previous study from Sweden [21]. Second, this population-based study, representative of the general population, had a satisfactory sample size of randomly invited adults from the HUNT4 survey, all undergoing a systematic full-mouth clinical examination. The study population is heterogeneous in terms of age, sex, health status, socioeconomic status, and lifestyle factors. This allows us to investigate the association between a range of exposures and OMLs. Third, analyzing both the overall and specific types of OMLs on individual-level data and their distributions across age groups in an adult population highlights a key strength of the present study. Fourth, a review by da Silva et al. [7] suggested that many previous studies estimating OMLs prevalence have methodological weaknesses, such as not reporting response rates, lacking standardization, omitting reasons for excluding individuals from study, and presenting results without appropriate confidence intervals, making comparisons across studies challenging in certain populations. However, these issues are addressed in our study.

This study also has some limitations. First, the data of this study was collected from individuals living in Nord-Trøndelag County, representing smaller cities and rural areas, and may not fully capture more diverse community settings. Although the HUNT population is representative of the Norwegian population, its relatively homogenous population may restrict the generalizability of the findings to other populations [7, 25]. Additionally, self-selection and non-response bias may have influenced our study findings, potentially limiting the generalizability of the results to other populations. Second, data on the history of underlying medical diseases were self-reported which may confer limited predictive accuracy due to potential inaccuracies in reporting. Third, most studies that incorporate biopsies do so primarily to exclude malignancy [34–37]. Our study did not include histopathological verification of clinical diagnoses of OMLs. While histopathology would enhance diagnostic accuracy, it may be impractical in large-scale epidemiological studies due to resource constraints. Fourth, while we did not perform an inter-examiner reliability test, oral mucosal photos with lesions were carefully evaluated by a specialist (TRK) and reassessed by another specialist (LMB) when needed to ensure diagnostic accuracy. This approach enhances the validity of our findings and reduces potential bias, thereby strengthening the findings of the study. Fifth, the cross-sectional design of this study captures data at a single point in time, which limits the ability to establish temporal associations between various exposure(s) and OMLs. Lastly, the limited statistical power restricted our ability to investigate the association between exposures and certain OML types. Also, it is important to note that wide confidence intervals indicate greater uncertainty in these point estimates, and as such, the results should be interpreted with caution.

## Conclusion

This population-based cross-sectional study found an OMLs prevalence of 7.5% among Norwegian adults, with higher prevalence in those with older age. This study found associations between some exposures and OML and its types. Larger studies in a similar population, examining the associations between socioeconomic, lifestyle, and clinical factors with OMLs and their types are needed to replicate our findings.

### Implication

From a clinical and public health perspective, this study shows the prevalence and distribution of OML’s in different age groups and selected exposures, which is important knowledge for both general dentists and primary care physicians.

## Electronic supplementary material

Below is the link to the electronic supplementary material.

Supplementary Material 1

## Data Availability

The data which this study is based on are housed in the HUNT databank, and biological samples are stored in the HUNT biobank. The HUNT Research Centre is authorized by the Norwegian Data Inspectorate to manage and store these data. The primary identification method in the database is the personal identification number assigned to all Norwegians and residents of Norway at birth or immigration. De-identified data are provided to scientists upon approval of a research protocol by the Regional Ethical Committee and the HUNT Research Centre. To ensure participant privacy, the HUNT Research Centre restricts data storage outside the HUNT databank and does not deposit data in open repositories. Detailed information on exported data is available within the HUNT databank and can be reproduced upon request. There are no restrictions on data export with approved applications to the HUNT Research Centre. Sharing data from this study will be facilitated by the corresponding author upon reasonable request, contingent upon approval from the HUNT Research Centre. For further details, visit www.ntnu.edu/hunt/data.

## Abbreviations

BMI: Body Mass Index
CRP: C-reactive protein
CI: Confidence interval
CVD: Cardiovascular diseases
COPD: Chronic obstructive pulmonary disease
DAG: Directed acyclic graph
HUNT: The Trøndelag Health Study
NOK: Norwegian Kroner
NHANES III: Third National Health and Nutrition Examination Survey
OMLs: Oral mucosal lesions
OR: Odds ratios
SD: Standard deviation
TkMidt: The Center for Oral Health Services and Research
WHO: World Health Organization
