# Supplementary material for: Prevalence of oral mucosal lesions and associated risk factors in a Norwegian adult population – the HUNT4 Oral Health study
Source: BMC Oral Health. 2025 Jul 4;25:1099. doi: 10.1186/s12903-025-06162-4 (PMC12228229; doi:10.1186/s12903-025-06162-4)
Supplement: Supplementary file 1 — Supplementary Material 1 [file 12903_2025_6162_MOESM1_ESM.docx]

**Supplementary Information**

Supplementary Table S1 Distribution and proportion of history of major chronic diseases in the study population

Supplementary Table S2 Frequency of OMLs diagnosed across different age groups in the study population.

Supplementary Table S3 Multivariable logistic regression analyses were performed to assess the association between exposure(s) and the presence of exophytic OML lesion types.

Supplementary Table S4 Multivariable logistic regression analyses were performed to assess the association between exposure(s) and the presence of white OML lesion types.

Supplementary Table S5 Multivariable logistic regression analyses were performed to assess the association between exposure(s) and the presence of red-blue OML lesion types.

Supplementary Table S6 Multivariable logistic regression analyses were performed to assess the association between exposure(s) and the presence of red OML lesion types.

**Table S1 Distribution and Proportion of history of major chronic diseases in the study population**

| **History of Chronic Diseases** | **N (%)** |
| --- | --- |
| **Cancer ª**  **Yes**  Unknown | 332 (6.8)  168 (3.4) |
| **CVDᵇ**  **Yes**  Unknown | 514 (10.5)  211 (4.3) |
| **Rheumatic diseasesᶜ**  **Yes**  Unknown | 517 (10.5)  210 (4.3) |
| **Respiratory diseasesᵈ**  **Yes**  Unknown | 654 (13.3)  184 (3.7) |
| **Psychiatric disordersᵉ**  **Yes**  Unknown | 909 (18.5)  179 (3.6) |
| **Endocrine diseasesᶠ**  **Yes**  Unknown | 549 (11.2)  196 (4.0) |

ª Cancer history includes any kind of cancer history

ᵇ CVD= Cardiovascular disease history includes angina or heart attack or heart failure or atrial fibrillation or stroke

ᶜ Rheumatic diseases history includes psoriasis or arthritis or ankylosing spondylitis

ᵈ Respiratory diseases history includes asthma or COPD= chronic obstructive pulmonary disease

ᵉ Psychiatric disorders history includes any kind of psychiatric disorders

ᶠ Endocrine diseases history includes any kind of diabetes or any kind of thyroid disorders

Unknown = missing data

**Table S2 Frequency of OMLs diagnosed across different age groups in the study population.**

|  | Total number of lesions | **Age Distribution**  (in years) | | | |
| --- | --- | --- | --- | --- | --- |
|  |  | 19-39 | 40-59 | 60-79 | ≤80 |
| **Distribution of lesions in groups** | **412^a^** |  |  |  |  |
| **Exophytic lesions**  -Fibroma (D10.30)  - Papilloma (D10.30)  - Fibrous Epulis (K06.8)  - Mucocele-oral (K11.6)  - Fordyce granules (Q38.6)  - Lipoma oral (D17.0) | **157**  89  22  3  18  23  2 | 15  3  2  1  2  6  1 | 57  39  4  0  2  12  0 | 73  42  12  1  13  4  1 | 12  5  4  1  1  1  0 |
| **White lesions**  - Leucoplakia (K13.2)  - Oral Lichen Planus (L43.9)  (Oral mucosal lichen planus)  - Leukokeratosis nicotina palati  (Smoker's palate) (K13.2)  or Nicotine stomatitis (K13.2)  - Morsicatio buccarum  (Chronic cheek biting) (K13.1) | **76**  33  30  1  12 | 11  7  2  1  1 | 24  10  8  0  6 | 34  12  17  0  5 | 7  4  3  0  0 |
| **Red-blue lesions**  - Oral Hemangiomas (D18.09)  or Hemangioma lip/skin (D18.01) | **63** | 0 | 16 | 41 | 6 |
| **Red lesions**  - Erythroplakia (K13.2)  - mucous membrane pemphigoid (L12.1)  - Rhagades and cheilitis (K13.0) | **42**  30  1  11 | 8  5  0  3 | 19  12  1  6 | 12  11  0  1 | 3  2  0  1 |
| **Tongue Lesions**  -Lingua villosa nigra (K14.3)  -Lingua plicata (K14.5)  -Lingua geografica (K14.1)  -Median rhomboid glossitis (K14.2) | **33**  4  1  24  4 | 7  0  0  6  1 | 13  2  1  9  1 | 12  2  0  8  2 | 1  0  0  1  0 |
| **Ulcerative lesions**  - Herpes labialis (B00.1)  or Aphthous ulcerations (K12.0)  or Aphthous Stomatitis (K12.0)  - Long period ulceration (K12.30) | **30**  25  5 | 8  7  1 | 14  13  1 | 7  5  2 | 1  0  1 |
| **Other alteration**  - Lentigo (L81.4)  or Nevus - Melanocytic naevi of lip (D22.0)  or Solar elastosis (L57.9) | **11** | 4 | 3 | 4 | 0 |

^a^Number of oral mucosal lesions observed among individuals being diagnosed (n=367)

**Table S3 Multivariable logistic regression analyses were performed to assess the association between exposure(s) and the presence of exophytic OML lesion types.**

| **Exposure(s)** | **Age-adjusted**  **Model 1**  OR (95% CI) | **Fully-adjusted**  **Model 2**  OR (95% CI) |
| --- | --- | --- |
| **Education level^a^**  High  Middle/Low | N=4793  1.00(ref.)  1.06 (0.76-1.48) | N=4793  1.00(ref.)  1.10 (0.77-1.56) |
| **Household income^b^**  Very high  High  Middle  Low  Very low | N=4793  1.00(ref.)  1.05 (0.60-1.82)  1.49 (0.91-2.44)  0.93 (0.53-1.65)  0.63 (0.25-1.57) | N=4793  1.00(ref.)  1.02 (0.58-1.79)  1.45 (0.87-2.40)  0.90 (0.50-1.65)  0.63 (0.24-1.62) |
| **Visit to Dentist^d^**  No  Yes | N=3956  1.00(ref.)  0.85 (0.49-1.48) | N=3956  1.00(ref.)  0.80 (0.46-1.41) |
| **Smoking^d^**  Never  Ever | N=4777  1.00(ref.)  0.89 (0.64-1.24) | N=4777  1.00(ref.)  0.87(0.62-1.21) |
| **Snus use^e^**  Never  Ever | N=4734  1.00(ref.)  1.34 (0.88-2.04) | N=4734  1.00(ref.)  1.33 (0.85-2.10) |
| **BMI categories^d^, Kg/m^2^**  Healthy  Overweight  Obese | N=4744  1.00(ref.)  1.20 (0.81-1.77)  1.15 (0.73-1.82) | N=4744  1.00(ref.)  1.17 (0.79-1.73)  1.13 (0.72-1.80) |
| **Dental Health Status^f^**  Very Good  Good  Bad  Very bad | N=3513  1.00(ref.)  1.11 (0.67-1.85)  0.61 (0.26-1.41)  0 | N=3513  1.00(ref.)  1.1 (0.66-1.84)  0.63 (0.27-1.48)  0 |
| **General Health Status^g^**  Very Good  Good  No so Good  Poor | N=4733  1.00  1.05 (0.65-1.69)  0.67 (0.37-1.21)  1.21 (0.28-5.34) | N=4733  1.00(ref.)  1.06 (0.65-1.71)  0.70 (0.38-1.28)  1.26 (0.28-5.60) |
| **Cancer^g^**  No  Yes | N=4631  1.00(ref.)  1.24 (0.73-2.09) | N=4631  1.00(ref.)  1.24 (0.73-2.1) |
| **CVD^g^**  No  Yes | N=4587  1.00(ref.)  1.22 (0.77-1.93) | N=4587  1.00(ref.)  1.22 (0.77-1.94) |
| **Rheumatic Diseases^c^**  No  Yes | N=4596  1.00(ref.)  0.92 (0.55-1.52) | N=4596  1.00(ref.)  0.86 (0.51-1.45) |
| **Respiratory Diseases^g^**  No  Yes | N=4613  1.00(ref.)  1.07 (0.66-1.73) | N=4613  1.00(ref.)  1.09 (0.67-1.78) |
| **Psychiatric Disorders^g^**  No  Yes | N=4621  1.00(ref.)  1.18 (0.78-1.79) | N=4621  1.00(ref.)  1.24 (0.81-1.90) |
| **Endocrine Diseases^g^**  No  Yes | N=4604  1.00(ref.)  0.60 (0.34-1.05) | N=4604  1.00(ref.)  0.63 (0.36-1.12) |
| **Multimorbidity^g^**  0  1  ≥2 | N=4387  1.00(ref.)  1.03 (0.70-1.52)  0.98 (0.61-1.58) | N=4387  1.00(ref.)  1.05 (0.71-1.55)  1.03 (0.64-1.68) |

Model 2 (Fully-adjusted model includes confounders presented below)

^a^ **Education level**: adjusted for age, income

^b^ **Household income:** adjusted for age, education

^c^ **Rheumatic diseases:** adjusted for age, sex, CRP

^d^ **Visit to dentist, smoking, BMI level:** adjusted for age, sex, education, income.

^e^ **Snus use:** adjusted for age, sex, education, income, smoking (never, ever)

^f^ **Dental health status:** adjusted for age, sex, education, income, smoking (never, ever), frequency of toothbrushing.

^g^ **General health status, Cancer history, CVD, Respiratory Diseases, Psychiatric Disorders, Endocrine diseases, Multimorbidity:** adjusted for age, sex, education, income, smoking (never, ever).

**Table S4 Multivariable logistic regression analyses were performed to assess the association between exposure(s) and the presence of white OML lesion types.**

| **Exposure(s)** | **Age-adjusted**  **Model 1**  OR (95% CI) | **Fully-adjusted**  **Model 2**  OR (95% CI) |
| --- | --- | --- |
| **Education level^a^**  High  Middle/Low | N=4793  1.00(ref.)  1.32 (0.81-2.16) | N=4793  1.00(ref.)  1.30 (0.78-2.19) |
| **Household income^b^**  Very high  High  Middle  Low  Very low | N=4793  1.00(ref.)  1.24 (0.57-2.72)  1.40 (0.68-2.90)  1.18 (0.53-2.66)  1.27 (0.43-3.71) | N=4793  1.00(ref.)  1.16 (0.53-2.56)  1.29 (0.61-2.72)  1.09 (0.47-2.53)  1.26 (0.42-3.84) |
| **Visit to Dentist^d^**  No  Yes | N=3956  1.00(ref.)  1.67 (0.60-4.64) | N=3956  1.00(ref.)  1.65 (0.59-4.65) |
| **Smoking^d^**  Never  Ever | N=4777  1.00(ref.)  1.80 (1.08-3.02) | N=4777  1.00(ref.)  1.77 (1.05-2.97) |
| **Snus use^e^**  Never  Ever | N=4734  1.00(ref.)  1.64 (0.94-2.88) | N=4734  1.00(ref.)  1.47 (0.79-2.74) |
| **BMI categories^d^, Kg/m^2^**  Healthy  Overweight  Obese | N=4744  1.00(ref.)  0.99 (0.56-1.75)  1.40 (0.76-2.59) | N=4744  1.00(ref.)  0.96 (0.54-1.70)  1.35 (0.73-2.50) |
| **Dental Health Status^f^**  Very Good  Good  Bad  Very bad | N=3513  1.00(ref.)  1.11 (0.53-2.30)  0.83 (0.27-2.52)  0 | N=3513  1.00(ref.)  1.06 (0.51-2.22)  0.80 (0.26-2.46)  0 |
| **General Health Status^g^**  Very Good  Good  No so Good  Poor | N=4733  1.00(ref.)  1.24 (0.60-2.58)  1.03 (0.44-2.44)  6.55 (1.94-22.01) | N=4733  1.00(ref.)  1.14 (0.54-2.39)  0.92 (0.38-2.23)  5.94 (1.72-20.56) |
| **Cancer^g^**  No  Yes | N=4631  1.00(ref.)  0.71 (0.28-1.82) | N=4631  1.00(ref.)  0.71 (0.28-1.81) |
| **CVD^g^**  No  Yes | N=4587  1.00(ref.)  0.80 (0.38-1.67) | N=4587  1.00(ref.)  0.78 (0.37-1.64) |
| **Rheumatic Diseases^c^**  No  Yes | N=4596  1.00(ref.)  0.93 (0.44-1.96) | N=4596  1.00(ref.)  0.84 (0.40-1.79) |
| **Respiratory Diseases^g^**  No  Yes | N=4613  1.00(ref.)  0.86 (041-1.81) | N=4613  1.00(ref.)  0.83 (.039-1.75) |
| **Psychiatric Disorders^g^**  No  Yes | N=4621  1.00(ref.)  1.53 (0.87-2.66) | N=4621  1.00(ref.)  1.48 (0.84-2.62) |
| **Endocrine Diseases^g^**  No  Yes | N=4604  1.00(ref.)  0.93 (0.45-1.91) | N=4604  1.00(ref.)  0.91 (0.44-1.88) |
| **Multimorbidity^g^**  0  1  ≥2 | N=4387  1.00(ref.)  1.26 (0.73-2.17)  1.10 (0.54-2.24) | N=4387  1.00(ref.)  1.21 (0.70-2.10)  1.06 (0.52-2.17) |

Model 2 (Fully-adjusted model includes confounders presented below)

^a^ **Education level**: adjusted for age, income

^b^ **Household income:** adjusted for age, education

^c^ **Rheumatic diseases:** adjusted for age, sex, CRP

^d^ **Visit to dentist, smoking, BMI level:** adjusted for age, sex, education, income.

^e^ **Snus use:** adjusted for age, sex, education, income, smoking (never, ever)

^f^ **Dental health status:** adjusted for age, sex, education, income, smoking (never, ever), frequency of toothbrushing.

^g^ **General health status, Cancer history, CVD, Respiratory Diseases, Psychiatric Disorders, Endocrine diseases, Multimorbidity:** adjusted for age, sex, education, income, smoking (never, ever).

**Table S5 Multivariable logistic regression analyses were performed to assess the association between exposure(s) and the presence of red-blue OML lesion types.**

| **Exposure(s)** | **Age-adjusted**  **Model 1**  OR (95% CI) | **Fully-adjusted**  **Model 2**  OR (95% CI) |
| --- | --- | --- |
| **Education level^a^**  High  Middle/Low | N=4793  1.00(ref.)  1.06 (0.63-1.78) | N=4793  1.00(ref.)  1.06 (0.61-1.83) |
| **Household income^b^**  Very high  High  Middle  Low  Very low | N=4793  1.00(ref.)  1.79 (0.72-4.43)  1.13 (0.46-2.80)  1.07 (0.41-2.82)  1.69 (0.55-5.22) | N=4793  1.00(ref.)  1.76 (0.71-4.38)  1.10 (0.44-2.78)  1.06 (0.39-2.90)  1.75 (0.54-5.72) |
| **Visit to Dentist^d^**  No  Yes | N=3956  1.00(ref.)  0.73 (0.31-1.74) | N=3956  1.00(ref.)  0.69 (0.28-1.67) |
| **Smoking^d^**  Never  Ever | N=4777  1.00(ref.)  0.73 (0.44-1.21) | N=4777  1.00(ref.)  0.74 (0.45-1.23) |
| **Snus use^e^**  Never  Ever | N=4734  1.00(ref.)  1.03 (0.49-2.15) | N=4734  1.00(ref.)  1.09 (0.50-2.40) |
| **BMI categories^d^, Kg/m^2^**  Healthy  Overweight  Obese | N=4744  1.00(ref.)  1.13 (0.64-2.02)  0.92 (0.45-1.89) | N=4744  1.00(ref.)  1.10 (0.61-1.97)  0.81 (0.44-1.87) |
| **Dental Health Status^f^**  Very Good  Good  Bad  Very bad | N=3513  1.00(ref.)  0.61 (0.31-1.19)  0.65 (0.24-1.78)  1.16 (0.15-9.32) | N=3513  1.00(ref.)  0.64 (0.33-1.28)  0.77 (0.27-2.15)  1.41 (0.17-11.47) |
| **General Health Status^g^**  Very Good  Good  No so Good  Poor | N=4733  1.00(ref.)  0.60 (0.31-1.17)  0.52 (0.23-1.15)  1.01 (0.13-8.05) | N=4733  1.00(ref.)  0.61 (0.31-1.21)  0.53 (0.23-1.22)  1.00 (0.12-8.16) |
| **Cancer^g^**  No  Yes | N=4631  1.00(ref.)  2.13 (1.13-4.02) | N=4631  1.00(ref.)  2.17 (1.14-4.11) |
| **CVD^g^**  No  Yes | N=4587  1.00(ref.)  0.80 (0.40-1.60) | N=4587  1.00(ref.)  0.78 (0.38-1.59) |
| **Rheumatic Diseases^c^**  No  Yes | N=4596  1.00(ref.)  1.74 (0.95-3.21) | N=4596  1.00(ref.)  1.84 (0.99-3.40) |
| **Respiratory Diseases^g^**  No  Yes | N=4613  1.00(ref.)  1.00 (0.47-2.13) | N=4613  1.00(ref.)  1.03 (0.48-2.20) |
| **Psychiatric Disorders^g^**  No  Yes | N=4621  1.00(ref.)  0.74 (0.35-1.57) | N=4621  1.00(ref.)  0.77 (0.36-1.64) |
| **Endocrine Diseases^g^**  No  Yes | N=4604  1.00(ref.)  0.54 (0.23-1.27) | N=4604  1.00(ref.)  0.56 (0.23-1.32) |
| **Multimorbidity^g^**  0  1  ≥2 | N=4387  1.00(ref.)  0.66 (0.35-1.24)  1.02 (0.53-1.96) | N=4387  1.00(ref.)  0.66 (0.35-1.25)  1.05 (053-2.05) |

Model 2 (Fully-adjusted model includes confounders presented below)

^a^ **Education level**: adjusted for age, income

^b^ **Household income:** adjusted for age, education

^c^ **Rheumatic diseases:** adjusted for age, sex, CRP

^d^ **Visit to dentist, smoking, BMI level:** adjusted for age, sex, education, income.

^e^ **Snus use:** adjusted for age, sex, education, income, smoking (never, ever)

^f^ **Dental health status:** adjusted for age, sex, education, income, smoking (never, ever), frequency of toothbrushing.

^g^ **General health status, Cancer history, CVD, Respiratory Diseases, Psychiatric Disorders, Endocrine diseases, Multimorbidity:** adjusted for age, sex, education, income, smoking (never, ever).

**Table S6 Multivariable logistic regression analyses were performed to assess the association between exposure(s) and the presence of red OML lesion types.**

| **Exposure(s)** | **Age-adjusted**  **Model 1**  OR (95% CI) | **Fully-adjusted**  **Model 2**  OR (95% CI) |
| --- | --- | --- |
| **Education level^a^**  High  Middle/Low | N=4793  1.00(ref.)  1.45 (0.74-2.81) | N=4793  1.00(ref.)  1.52 (0.75-3.11) |
| **Household income^b^**  Very high  High  Middle  Low  Very low | N=4793  1.00(ref.)  0.55 (0.2-1.53)  0.75 (0.31-1.81)  1.16 (0.47-2.84)  0.60 (0.13-2.74) | N=4793  1.00(ref.)  0.50 (0.18-1.41)  0.65 (0.26-1.63)  0.99 (0.38-2.56)  0.53 (0.11-2.54) |
| **Visit to Dentist^d^**  No  Yes | N=3956  1.00(ref.)  2.24 (0.52-9.56) | N=3956  1.00(ref.)  2.19 (0.51-9.49) |
| **Smoking^d^**  Never  Ever | N=4777  1.00(ref.)  1.03 (0.54-1.98) | N=4777  1.00(ref.)  1.01 (0.52-1.95) |
| **Snus use^e^**  Never  Ever | N=4734  1.00(ref.)  0.67 (0.29-1.59) | N=4734  1.00(ref.)  0.74 (0.29-1.87) |
| **BMI categories^d^, Kg/m^2^**  Healthy  Overweight  Obese | N=4744  1.00(ref.)  1.12 (0.52-2.39)  1.28 (0.55-2.99) | N=4744  1.00(ref.)  1.20 (0.56-2.57)  1.30 (0.56-3.05) |
| **Dental Health Status^f^**  Very Good  Good  Bad  Very bad | N=3513  1.00(ref.)  2.88 (0.67-12.35)  2.68 (0.45-16.18)  0 | N=3513  1.00(ref.)  2.70 (0.62-11.72)  2.45 (0.39-15.27)  0 |
| **General Health Status^g^**  Very Good  Good  No so Good  Poor | N=4733  1.00(ref.)  0.90 (0.40-2.04)  0.65 (0.22-1.92)  0 | N=4733  1.00(ref.)  0.85 (0.37-1.94)  0.56 (0.18-1.72)  0 |
| **Cancer^g^**  No  Yes | N=4631  1.00(ref.)  1.15 (0.34-3.89) | N=4631  1.00(ref.)  1.16 (0.34-3.95) |
| **CVD^g^**  No  Yes | N=4587  1.00(ref.)  0.77 (0.22-2.64) | N=4587  1.00(ref.)  0.85 (0.24-2.95) |
| **Rheumatic Diseases^c^**  No  Yes | N=4596  1.00(ref.)  0.75 (0.23-2.49) | N=4596  1.00(ref.)  0.94 (0.33-2.67) |
| **Respiratory Diseases^g^**  No  Yes | N=4613  1.00(ref.)  0.78 (0.28-2.22) | N=4613  1.00(ref.)  0.80 (0.28-2.27) |
| **Psychiatric Disorders^g^**  No  Yes | N=4621  1.00(ref.)  0.99 (0.43-2.25) | N=4621  1.00(ref.)  0.93 (0.40-2.16) |
| **Endocrine Diseases^g^**  No  Yes | N=4604  1.00(ref.)  1.92 (0.82-4.51) | N=4604  1.00(ref.)  1.85 (0.78-4.38) |
| **Multimorbidity^g^**  0  1  ≥2 | N=4387  1.00(ref.)  0.80 (0.37-1.76)  1.09 (0.42-2.84) | N=4387  1.00(ref.)  0.80 (0.36-1.74)  1.07 (0.41-2.83) |

Model 2 (Fully-adjusted model includes confounders presented below)

^a^ **Education level**: adjusted for age, income

^b^ **Household income:** adjusted for age, education

^c^ **Rheumatic diseases:** adjusted for age, sex, CRP

^d^ **Visit to dentist, smoking, BMI level:** adjusted for age, sex, education, income.

^e^ **Snus use:** adjusted for age, sex, education, income, smoking (never, ever)

^f^ **Dental health status:** adjusted for age, sex, education, income, smoking (never, ever), frequency of toothbrushing.

^g^ **General health status, Cancer history, CVD, Respiratory Diseases, Psychiatric Disorders, Endocrine diseases, Multimorbidity:** adjusted for age, sex, education, income, smoking (never, ever).
